# Is Balance Training Using the Stabilometric Platforms Integrating Virtual Reality and Feedback Effective for Patients with Non-Diabetic Peripheral Neuropathy?—A Systematic Review

**DOI:** 10.3390/jcm14228049

**Published:** 2025-11-13

**Authors:** Diana-Maria Stanciu, Oana-Georgiana Cernea, Laszlo Irsay, Viorela-Mihaela Ciortea, Mădălina-Gabriela Iliescu, Mihaela Stanciu, Florina-Ligia Popa

**Affiliations:** 1 Doctoral School, Faculty of Medicine, “Lucian Blaga” University of Sibiu, 550169 Sibiu, Romania; diana.rusu@ulbsibiu.ro (D.-M.S.); oanageorgiana.spatariu@ulbsibiu.ro (O.-G.C.); 2 Department of Rehabilitation Medicine, “Iuliu Hatieganu” University of Medicine and Pharmacy, 400012 Cluj-Napoca, Romania; laszlo.irsay@umfcluj.ro (L.I.); viorela.ciortea@umfcluj.ro (V.-M.C.); 3 Department of Physical Medicine and Rehabilitation, Faculty of Medicine, “Ovidius” University of Constanta, 900470 Constanta, Romania; madalina.iliescu@365.univ-ovidius.ro; 4 Department of Endocrinology, Faculty of Medicine, “Lucian Blaga” University of Sibiu, 550169 Sibiu, Romania; mihaela.stanciu@ulbsibiu.ro; 5 Department of Physical Medicine and Rehabilitation, Faculty of Medicine, “Lucian Blaga” University of Sibiu, 550024 Sibiu, Romania

**Keywords:** peripheral neuropathy, balance training, stabilometric platform, virtual reality, neurorehabilitation

## Abstract

**Background**: Peripheral neuropathy (PN) refers to a spectrum of symptoms resulting from dysfunctions of the peripheral sensory, motor, and autonomic neurons. PN is associated with significant balance impairments and an increased risk of falls, contributing to reduced functional independence and quality of life. Although diabetic PN has been extensively investigated, there remains a lack of synthesized evidence regarding rehabilitation approaches for individuals with non-diabetic PN. This systematic review aims to evaluate the effectiveness of stabilometric platforms incorporating virtual reality (VR) and feedback (FB) in improving balance and related outcomes in patients with PN of various etiologies. **Methods**: This systematic review was conducted in accordance with the Preferred Reporting Items for Systematic Reviews and Meta-Analyses (PRISMA) guidelines and the protocol registered in PROSPERO (CRD420251086625). Seven major databases (PubMed, Scopus, ScienceDirect, Cochrane, Web of Science, Springer, and Wiley) were searched from inception to April 2025. Studies including adult patients with non-diabetic PN undergoing balance rehabilitation using stabilometric platforms with VR and FB were considered. The methodological quality of the included studies was assessed using the PEDro scale, RoB2, and ROBINS-I V2 tools. **Results**: A total of six studies met the inclusion criteria, encompassing 133 participants with non-diabetic PN. Interventions involving specialized balance training platforms incorporating VR and FB demonstrated significant improvements in both static and dynamic balance and postural control, as well as a reduction in the risk of falling. These systems also showed favorable adherence rates to rehabilitation programs. However, variability in intervention protocols and outcome measures limited the ability to perform direct comparisons across studies. **Conclusions:** The use of stabilometric platforms appears to be a promising approach for balance rehabilitation in patients with non-diabetic PN. Despite the limited number of included studies, the results support their integration into rehabilitation programs for this patient population. Further large-scale, high-quality studies are needed to establish standardized protocols and confirm long-term efficacy.

## 1. Introduction

Peripheral neuropathy (PN) represents a complex spectrum of neurological disorders characterized by damage to peripheral nerves, leading to significant functional impairments [1,2]. It constitutes a public health concern, particularly among the elderly, with an annual incidence of 77 cases per 100,000 individuals and a prevalence ranging from 1% to 12% across all age groups, reaching up to 8% in older adults [3,4].

Although the most widely recognized and studied form of PN is diabetic peripheral neuropathy (DPN), numerous other types affect millions of individuals worldwide, including chemotherapy-induced, idiopathic, and hereditary variants [5]. Other common etiologies include chronic alcohol use, toxic exposures, hereditary motor–sensory neuropathies (HMSN), paraproteinemias, chronic kidney disease, and nutritional deficiencies [6]. There are also rare forms of PN, such as Guillain–Barré syndrome (GBS), chronic inflammatory demyelinating polyneuropathy (CIDP), and multifocal motor neuropathy (MMN), which carry significant clinical relevance due to their requirement for distinct diagnostic approaches and potentially differentiated therapeutic strategies [7]. These conditions typically manifest through progressive sensory loss, muscle weakness, and impaired proprioception, ultimately compromising balance control and increasing the risk of falls and fractures [8,9,10].

In clinical and research contexts, balance performance is typically assessed through validated functional and instrumented measures that capture both static and dynamic postural control. Commonly used clinical tools include the Berg Balance Scale (BBS) [11], which quantifies functional balance through task-based performance; the Timed Up and Go (TUG) test [12], evaluating dynamic stability and mobility; and the Limits of Stability (LOS) test [13], which assesses the ability to voluntarily shift the center of gravity within the base of support. In addition, stabilometric assessments—performed using force or pressure platforms—enable the objective quantification of center-of-pressure (COP) displacement, sway area, path length, and velocity, providing sensitive indicators of postural steadiness and sensorimotor integration [14,15]. The combination of these functional and instrumental approaches enables a comprehensive evaluation of balance deficits and rehabilitation outcomes in patients with PN.

Current rehabilitation approaches for PN-related balance disorders primarily rely on conventional physiotherapy methods, including exercises targeting proprioception, muscle strengthening, and balance training [16,17]. However, these traditional interventions often demonstrate limited effectiveness in managing the complex sensory integration deficits characteristic of PN.

Recent advances in rehabilitation technology suggest promising alternatives, particularly through FB-based and virtual reality (VR) interventions, which have shown encouraging results in various neurological conditions such as stroke, Parkinson’s disease, and cerebral palsy [18]. Within this context, the integration of stabilometric platforms with VR and FB systems represents a sophisticated approach to balance rehabilitation.

In this review, the term „stabilometric platform” refers to a computer-assisted balance system equipped with force or pressure sensors that record postural sway and provide real-time visual, auditory, or tactile feedback to the user. Unlike traditional balance tools such as wobble boards or BOSU balls, these platforms offer precise quantification of COP parameters—including displacement, sway area, and velocity—and often integrate interactive software or immersive virtual environments to guide motor learning [14,15].

These platforms operate through multiple mechanisms: VR systems create controlled, immersive environments that enhance sensory integration and motor learning, while real-time FB is provided via visual, auditory, or tactile modalities is provided through visual, auditory, or tactile modalities to promote adaptive postural adjustments and motor control [18,19]. Although stabilometric systems can be used for both assessment and rehabilitative training, the present review specifically focuses on their therapeutic applications in patients with non-diabetic PN, where sensory loss and postural instability represent key targets of intervention.

Previous systematic reviews have extensively evaluated VR-based interventions in patients with DPN [18,20,21]; however, the broader spectrum of non-diabetic PN forms remains insufficiently explored. Therefore, this systematic review aimed to synthesize the current evidence on the effectiveness of stabilometric platforms integrating VR and FB on balance and gait outcomes in patients with non-diabetic PN.

## 2. Materials and Methods

This systematic review is based on the “Preferred Reporting Items for Systematic Reviews and Meta-Analysis” (PRISMA) methodology [22].

The review protocol was submitted and registered in the PROSPERO database under the registration number CRD420251086625.

### 2.1. The Search Strategy

In accordance with the PRISMA guidelines, our research included articles indexed in the following international databases: PubMed, Scopus, ScienceDirect, Web of Science, The Search Strategy Springer Nature Link, Cochrane Library, and Wiley Online Library. The search period was from inception until April 2025, including only articles in English.

To identify relevant studies, a systematic search was conducted using keywords such as “peripheral neuropathy”, “stabilometric platform”, “force platform”, “postural control”, “balance training”, “postural balance”, “rehabilitation”, and “physical therapy”, combined with operators “AND” and “OR”. The finalized Boolean search expression was (“peripheral neuropathy”) AND (“stabilometric platform” OR “force platform”) AND (“postural control” OR “balance training” OR “postural balance”) AND (“rehabilitation” OR “physical therapy”). In addition to the primary terms (‘stabilometric platform’, ‘force platform’), the search was broadened using alternative keywords such as ‘posturography’, ‘force plate’, ‘equilibrium training’, ‘center of pressure’, ‘virtual reality‘ and ‘feedback platform’ (added post hoc for transparency; no additional records were identified). Boolean combinations were adjusted across databases to capture variations in terminology. The complete search strings for each database are provided in Supplementary Table S1.

This systematic search strategy was designed to ensure a comprehensive yet focused retrieval of relevant literature, thereby supporting a methodologically rigorous review process. All identified articles were imported into Mendeley reference management software (Elsevier, Amsterdam, The Netherlands; latest version accessed on 15 September 2025, https://www.mendeley.com/) to facilitate organization and streamline the identification and removal of duplicates. The selection process was then managed using Rayyan (Qatar Computing Research Institute, Doha, Qatar) [23] to enable independent screening by two reviewers and to assist in blinding during the initial title and abstract review. The screening procedure was performed in stages, beginning with title and abstract review according to predefined inclusion and exclusion criteria, and followed by full-text assessment as part of the systematic review process.

### 2.2. The Study Selection

Two reviewers (D.-M.S. and O.-G.C.) independently screened the studies using predefined inclusion and exclusion criteria. The screening process consisted of an initial evaluation of titles and abstracts, followed by a full-text review of potentially eligible articles. Any disagreements were resolved through discussion until a consensus was reached. The screening process was not blinded to the authors of the studies or journals.

The Inclusion Criteria:

- Original research studies (including randomized controlled trials, cohort studies, case–control studies, cross-sectional and prospective observational studies, pilot trials, and case series) that investigate the use of platforms in the rehabilitative treatment of patients diagnosed with non-diabetic PN (e.g., CIDP, GBS, and CIPN) and balance impairments.
- Studies exploring the association between PN and balance-related deficits, such as impaired postural control, increased fall risk, or gait disturbances.
- Studies conducted on the adult population diagnosed with non-diabetic PN based on standardized or universally accepted diagnostic criteria (e.g., clinical examination, nerve conduction studies, validated neuropathy scales).
- Studies that report on outcomes related to balance performance, postural control, fall risk, or mobility impairments.
- Studies published in English with full-text availability.

The Exclusion Criteria:

- Studies that used stabilometric platforms exclusively for assessment or diagnostic purposes, without incorporating them into a therapeutic or rehabilitation intervention.
- Studies conducted on animals or participants under 18 years of age.
- Studies focusing exclusively on patients with DPN.
- Studies that do not evaluate balance-related outcomes.
- Reviews, systematic reviews, meta-analyses, conference abstracts, book chapters, study protocols, editorials, or letters to the editor.
- Non-English language publications or articles without full-text availability.

### 2.3. Data Extraction

Two reviewers independently performed data extraction using a standardized Excel form. Extracted data included study characteristics, participants, interventions, comparators, outcomes, and key findings. Any disagreements were resolved through discussion until a consensus was reached.

For each included study, the following data were extracted: first author, year of publication, country, study design, sample size, participants’ characteristics, type of PN, platform type and training program, frequency and duration, and outcome measures. The extracted information is summarized in Table 1 (study characteristics) and Table 2 (synthesis of results).

### 2.4. Quality Assessment

The methodological quality of the included studies was evaluated using three designated assessment tools. Two reviewers independently conducted the appraisal, and any discrepancies were resolved through discussion or, when necessary, by consulting a third reviewer.

Specifically, the Physiotherapy Evidence Database (PEDro) scal [24] was used for randomized controlled trials (maximum score 10 points; scores ≥6 considered high quality), while the Cochrane Risk of Bias 2 (RoB 2) tool [25] was applied to randomized studies to assess five domains: randomization process, deviations from intended interventions, missing outcome data, measurement of outcomes, and selective reporting. For non-randomized studies, the Risk Of Bias In Non-randomized Studies of Interventions, Version 2 (ROBINS-I V2) tool [26] was used to evaluate bias arising from confounding, selection, classification of interventions, deviations from intended interventions, missing data, measurement of outcomes, and reporting. The individual and summary assessments for each tool are described in detail in the Section 3.3.

### 2.5. Statistical Analysis

Because of the methodological diversity and variations in outcome reporting among the included studies, a quantitative meta-analysis could not be conducted. Instead, a narrative synthesis was performed to summarize and interpret the findings.

## 3. Results

**Figure 1:**
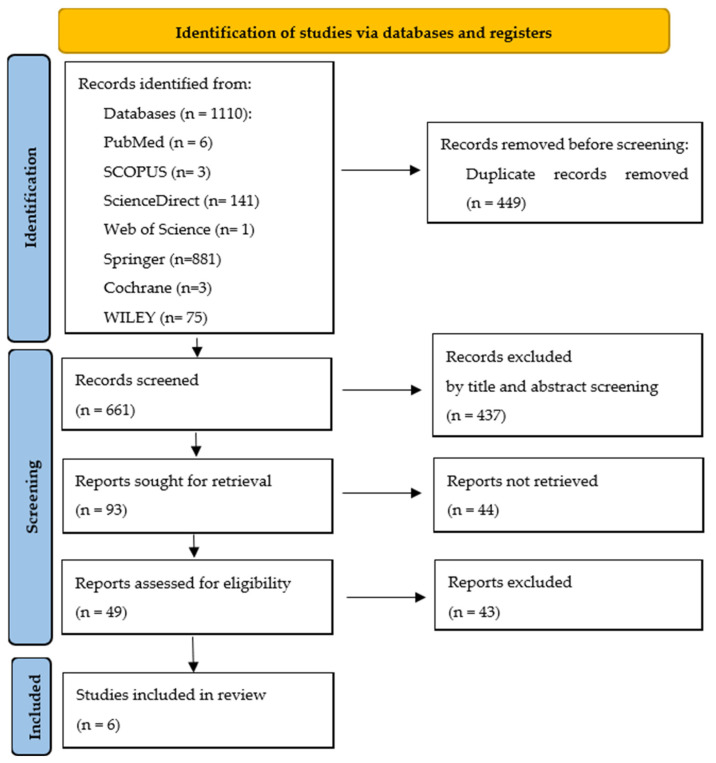
Flowchart of the study selection process according to PRISMA [22].

A total of 1110 studies were initially identified, as presented in Supplementary Table S1. Among the databases explored, Springer Nature Link yielded the highest number of results (881), followed by ScienceDirect with 141 records. Web of Science contributed a single result, while PubMed identified 6 relevant studies. Cochrane Library and Scopus provided a more limited number of studies, each contributing 3 results. These identified records formed the foundation for the subsequent screening and selection process. After duplicate removal, 661 articles remained. Screening of titles and abstracts led to the exclusion of 437 studies. Ninety-three full-text articles were assessed for eligibility. Of these, 44 reports could not be retrieved, as full-text versions were unavailable despite multiple retrieval attempts through institutional access or interlibrary requests. The remaining 43 articles were excluded after full-text review for the following reasons: ineligible population (e.g., DPN or healthy participants; *n* = 17), intervention not involving stabilometric or force platforms (*n* = 13), absence of balance or postural outcomes (*n* = 6), unsuitable study design such as reviews, conference abstracts, or case reports (*n* = 4), incomplete data reporting (*n* = 2), and overlapping datasets (*n* = 1). Finally, 6 studies met all inclusion criteria and were included in the qualitative synthesis. The complete selection process is illustrated in Figure 1 (PRISMA 2020 flow diagram) [22].

### 3.1. The Included Studies

A final selection of 6 studies met the predefined eligibility criteria and were ultimately included in this systematic review (Figure 1).

Table 1 provides an overview of the studies included in this review that employed various types of platforms for balance training in patients with PN. The table summarizes key information, including author and year of publication, country of origin, study design, sample size, and the number of references cited.

**Table 1:** Centralized clinical studies that have used different types of platforms for balance training in patients with non-diabetic PN.

Authors	Country	Year	Study Design	NO. Pac Incl	References
Cammisuli et al.	Italy	2016	non-randomized pilot study	7	[27]
Hosny et al.	Egypt	2022	single-blinded randomized comparative trial	30	[28]
Kerim et al.	Turkey	2018	randomized controlled trial	60	[29]
Nardone et al.	Italy	2010	crossover trial	33	[30]
Albiol-Pérez et al.	Spain	2013	case series	2	[31]
Hakim et al.	USA	2015	case report	1	[32]

### 3.2. The PICO Question

Eligibility for inclusion was based on the study’s relevance to the overarching research question of the systematic review: “Is balance training using the stabilometric platforms integrating VR and FB effective for patients with non-diabetic PN?”.

This research question was structured according to the PICO framework, as follows:

- P (Population): adults diagnosed with various types of PN, excluding those with DPN;
- I (Intervention): balance rehabilitation interventions utilizing stabilometric platforms;
- C (Comparison): conventional physiotherapy, absence of specific balance training, or no comparison group;
- O (Outcome): improvements in postural control, balance, or gait performance.

### 3.3. Risk of Bias

The methodological quality and risk of bias of the included studies were appraised using three validated tools, selected in accordance with the respective study designs.

For randomized controlled trials (RCTs), both the PEDro scale [24] and the RoB 2 tool [25] were employed.

The PEDro scale assesses internal validity across 11 items, including random allocation, concealed allocation, baseline comparability, blinding (of participants, therapists, and assessors), adequacy of follow-up, intention-to-treat analysis, between-group comparisons, and reporting of variability. While the first item is related to external validity and not scored, the remaining ten items contribute to a total score that reflects methodological rigor [24]. Table 2 shows the score on the PEDro scale [24] resulting from the 3 RTCs selected for this review. The overall score is 7.5 on average reflects the solid methodological quality of the selected studies.

**Table 2:** Score obtained on the PEDro scale [24] of the selected studies.

Study	Eligibility Criteria & Source (1)	Random Allocation (2)	Concealed Allocation (3)	Baseline Similarity (4)	Subject Blinding (5)	Therapist Blinding (6)	Assessor Blinding (7)	Adequate Follow-Up (8)	Intention-to-Treat (9)	Between-Group Statistical Comparisons (10)	Reporting of Point Estimates & Variability (11)	Total Score (2–11)
Hosny et al. [28]	yes	yes	yes	yes	no	no	yes	yes	yes	yes	yes	8/10
Kerim et al. [29]	yes	yes	yes	yes	yes	no	yes	yes	yes	yes	yes	9/10
Nardone et al. [30]	yes	yes	yes	no	no	no	no	yes	yes	yes	yes	6/10

Scoring: Scores 0–3 are considered ‘poor’, 4–5 ‘fair’, 6–8 ‘good’, and 9–10 ‘excellent’ [33].

**Figure 2:**
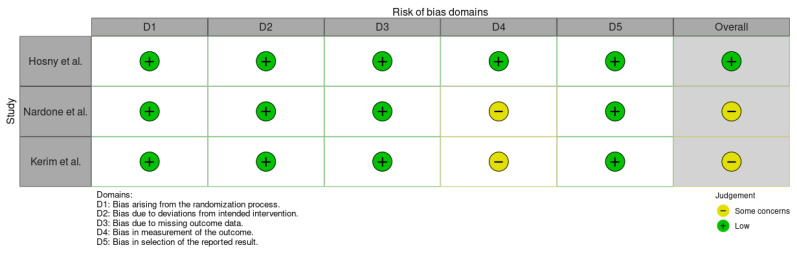
The risk of bias assessment using RoB 2 for the included studies [28,29,30]. Green = low risk; yellow = some concerns. Overall, the included RCTs demonstrated low risk across all domains, with minor concerns in outcome measurement (D4).

**Figure 3:**
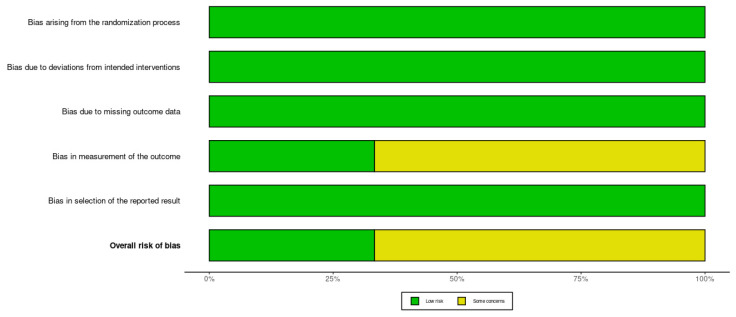
The summary visualization of RoB 2 ratings for the included studies. Bars represent the proportion of studies rated as low risk (green) or some concerns (yellow) in each domain. The majority of domains presented low risk, supporting good internal validity of the included RCTs.

The RoB 2 tool was additionally applied to provide a domain-based evaluation of bias across five dimensions: D1: bias arising from the randomization process; D2: bias due to deviations from intended interventions; D3: bias due to missing outcome data; D4: bias in measurement of the outcome; and D5: bias in selection of the reported result [25]. Each domain was rated as “low risk,” “some concerns,” or “high risk,” resulting in an overall judgment of the study’s risk of bias. Based on this assessment, among the RCTs, one study was rated as low risk of bias, and two were rated as having some concerns (Figure 2 and Figure 3). Overall, the quality of the included RCTs can be considered acceptable, though minor methodological limitations may affect the interpretability of some findings.

**Figure 4:**
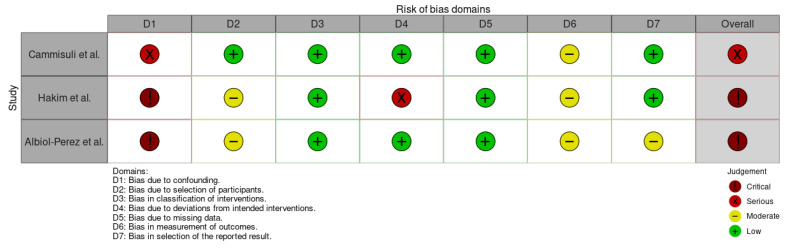
Domain-specific risk of bias assessment for the included non-randomized controlled trials using the ROBINS-I V2 tool [27,31,32]. Confounding (D1) and deviations from intended interventions (D4) were the domains most frequently associated with higher risk across studies.

**Figure 5:**
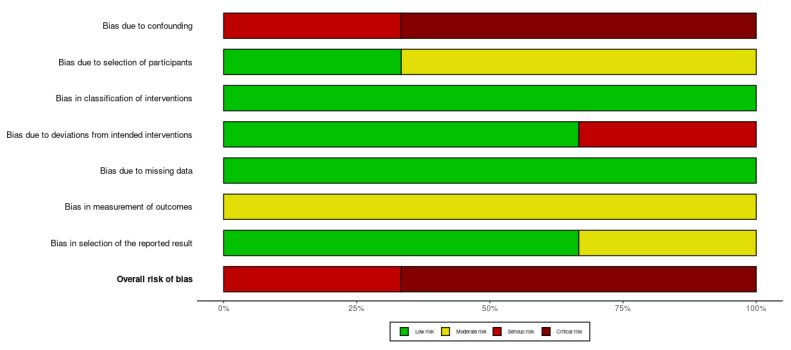
Overall summary visualization of ROBINS-I V2 ratings. Bars display the proportion of domains rated as low (green), moderate (yellow), serious, or critical (red) risk of bias. Confounding and intervention deviations were the primary sources of bias, while the overall risk of bias was rated as serious to critical across most studies.

For non-randomized studies, the ROBINS-I V2 tool [26] was used. This instrument evaluates potential bias across seven domains: D1: confounding; D2: selection of participants; D3: classification of interventions; D4: deviations from intended interventions; D5: missing data; D6: measurement of outcomes; and D7: selection of the reported result [26]. Each domain was judged as having a “low,” “moderate,” “serious,” or “critical” risk of bias, with a corresponding overall rating assigned to each study. The overall risk of bias across the non-randomized studies, as assessed using this tool, was judged to be either serious or critical, indicating substantial methodological limitations (Figure 4 and Figure 5). The most prominent weaknesses were the absence of any controlled comparison and the extremely small sample sizes, both of which significantly compromise the internal validity and generalizability of the findings. Consequently, the strength of the evidence derived from these studies is limited, and future research with more rigorous designs is needed to draw reliable conclusions.

All assessments were independently conducted by two reviewers. Any disagreements were resolved through discussion and consensus, with a third reviewer consulted in cases where consensus could not initially be reached.

### 3.4. Detailed Study Descriptions

A summary of the included studies’ main features is provided in Table 3.

Cammisuli et al. conducted a non-randomized pilot study in 2016 [27] that evaluated the use of a rehabilitation program based exclusively on visual computer-FB balance training (VCFBT) in patients diagnosed with chemotherapy-induced peripheral neuropathy (CIPN). The study included 7 patients aged 45 to 71 years who followed a rehabilitation program comprising 3 sessions of 60 min per week for 4 weeks using the Smart Balance Master (SBM). The VCFBT intervention led to significant improvements in several balance parameters. On the unstable surface with eyes open (EO), a notable reduction in center of gravity (COG) sway velocity was observed in 6 out of 7 patients (*p* = 0.004), while similar improvements, though not statistically significant, were seen with eyes closed (EC) (*p* = 0.057). On the stable surface, no significant changes in COG sway velocity were recorded. Additionally, VCFBT produced significant improvements across all parameters in the Limits of Stability (LOS) test. This therapy significantly enhanced the mean speed and step width in 6 out of 7 patients during the Tandem Walk (TW) test, although no effect was seen on End-sway. Furthermore, there was a significant improvement in the Berg Balance Scale (BBS) score in all patients post-therapy (*p* < 0.002). Although no significant effect was noted on step up/over (SUO), VCFBT led to a marked improvement in balance and mobility measures, particularly in COG sway velocity, LOS, and TW parameters, demonstrating its potential effectiveness in rehabilitation. The authors suggest the need for future larger studies in which VCFBT will be compared with standard physiotherapy.

Hosny et al. achieved in 2022 published a randomized comparative study [28] that evaluated the efficacy of game-like interactive exercise versus visual FB training on fall risk and sensory integration in patients with CIPN post-mastectomy. They included in their single-blind study 30 female patients, divided into two groups of 15 patients who followed a rehabilitation program three times per week for four weeks, 30 min/session. One group received game-like interactive exercises, and the other group received training with visual FB, all using the Biodex balance system. All the patients were evaluated using the modified total neuropathy score (mTNS) and the modified clinical test of sensory integration and balance (M-CTSIB). Post-treatment comparisons revealed a highly significant difference in the mean mTNS and risk of falling index (RFI) values between groups, favoring the group that received game-like exercises, while no significant difference was observed in the sway index of sensory integration (SI) (*p* = 0.174). The authors recommend further study of the combined and long-term effects of both methods.

Kerim et al. published a randomized controlled study in 2018 [29] involving 60 patients with peripheral neuropathic pain-related balance disorder. They were randomized into two groups of 30 patients; both received a 45 min individualized training session three times a week for 4 weeks. Balance exercises were given to all patients. At the same time, balance exercises were given with the Kinesthetic Ability Trainer (KAT) 2000 to the experimental group. In this study, both the balance exercise group and the KAT 2000 group showed significant improvements in static and dynamic scores assessed with the KAT 2000 system. However, the KAT 2000 group did not outperform the balance exercise group, and no statistically significant differences were found between the groups after four weeks of intervention. Similarly, no significant differences were observed between groups in the BBS or Timed Up and Go (TUG) scores, indicating no further improvement in dynamic balance with the combined approach. Regarding quality of life (QoL), both groups showed significant improvements across most domains, except for Nottingham Health Profile (NHP) sleep and energy levels in the balance exercise group. As only the short-term effects of balance exercise programs were analyzed, the authors highlight the need for further investigations to explore their long-term efficacy.

In 2010, Nardone et al. [30] assessed the efficacy of balance rehabilitation using a powered platform and specific exercises in 33 patients with vestibular disorders (*n* = 14) or PN (*n* = 19). This study aimed to assess whether powered platform training can improve balance control regardless of the underlying cause of impairment—neuropathy or vestibular disorders. Patients underwent both traditional balance exercises, focused on movement coordination rather than strength, and dynamic powered platform training. A crossover design was employed, allowing each patient to receive both interventions in reversed order, facilitating within-subject comparison of treatment effects. Seventeen patients first received training on the powered platform, followed by exercise (EX) therapy, while the remaining sixteen underwent the same treatments in reverse order. Exercise sessions were tailored, and patients with PN followed modified Frenkel exercises. All treatments were administered for one hour daily over a 10-day period, excluding weekends. Results of this study show that patients with neuropathy or vestibular deficits with balance problems improved postural control after a rehabilitation intervention based on both instrumental treatment (standing on an oscillating powered platform) and disease-specific balance EXs. Overall, improvement was greater in patients with vestibular disorders than in patients with neuropathy. It was not dependent on the order of the treatments; whether EXs preceded (EX–powered platform) or followed (powered platform–EX), the powered platform protocol did not make a difference. The improvement was evident regardless of the different indicators used (objective or clinical): sway area (SA) decreased, subjective score of stability increased, and evaluation scores of balance and gait improved.

A case series conducted by Albiol-Pérez et al. in 2013 used a virtual rehabilitation system (Active Balance Rehabilitation—ABAR) to increase the results in static and dynamic balance for Guillain–Barré patients [31]. The patients performed a total of 20 sessions using ABAR for 30 min each session and were evaluated based on static and dynamic balance. Both groups showed improvements in clinical test outcomes by the end of the intervention and at follow-up. Notable gains were observed in the Anterior Reach Test (ART), BBS, and Tinetti Test (TT). Additionally, the Suitability Evaluation Questionnaire (SEQ) test results indicated a high level of patient satisfaction.

A case report by Hakim et al., published in 2015 [32], described a 76-year-old patient with PN (unknown etiology) who followed for 6 weeks, 2 times a week, 1 h of Nintendo Wii Fit balance training sessions. The patient demonstrated notable improvements in balance and functional performance, even though Sensory Organization Test (SOT) values remained below normal levels. LOS showed a 22% increase in Maximum Endpoint Excursion (MXE), with normal reaction time and directional control. Motor Control Test (MCT) results improved in amplitude/scaling during forward translations, though latency remained delayed on both sides. Adaptation Test (ADT) scores also improved, particularly in response to downward platform rotations. Functional gains were reflected in higher BBS scores, better TUG performance, and increased repetitions in the 30 s Chair Stand Test (CST). Balance confidence, measured via the Activity-Specific Balance Confidence (ABC) scale, rose from 57.5% to 70.6%. The patient reported greater confidence and ease during walking and daily activities, both at home and in the community. Additionally, the patient expressed increased independence and enjoyment while using the Nintendo Wii Fit and reported no falls during the intervention or testing.

## 4. Discussion

This systematic review aimed to synthesize current evidence regarding the effects of platform-based training—integrating VR and FB—on balance and gait parameters in patients with non-diabetic PN. Overall, the interventions appear to improve outcomes related to balance and gait.

However, it must be emphasized that the results seem to be influenced by the variability of the treatment protocols employed. Moreover, not all studies assessed all relevant parameters, and there was considerable heterogeneity among the studies. Significant differences were noted regarding the type of devices used, the nature of the exercises, the duration and frequency of the interventions, as well as the design of the included studies. Additionally, the assessment methodologies were not standardized, particularly in terms of balance measurement, which was performed using diverse techniques and tools, thereby limiting the direct comparability of outcomes across studies.

The discussion is structured to sequentially address the main pathological aspects, the instruments and interventions applied, and the resulting outcomes related to balance, gait, fall risk, and quality of life. This thematic organization aims to facilitate a comprehensive understanding of the clinical implications of the reviewed evidence. The section concludes with a critical appraisal of the study’s methodological limitations and outlines future research directions to strengthen the evidence base for balance rehabilitation in non-diabetic PN.

### 4.1. Pathological Characteristics

All studies included in this systematic review focused on forms of non-diabetic PN, with varied etiologies, including immunological, toxic, iatrogenic, or idiopathic origins. For instance, the study by Albiol-Pérez et al. [31] included patients with GBS, an acute inflammatory demyelinating polyradiculoneuropathy of autoimmune nature, which primarily affects nerve roots and peripheral nerves, causing symmetrical muscle weakness and areflexia. At the other end of the spectrum, two other studies [27,28] focused on patients with CIPN, an acquired neurotoxic condition in which sensory and motor axonal damage is caused by chemotherapeutic agents such as taxanes or platinum-based compounds.

The patient cohort analyzed by Nardone et al. [30] included various forms of PN with diverse etiologies, spanning genetic, toxic, metabolic, autoimmune, and paraneoplastic spectrums. Charcot–Marie–Tooth (CMT) types 1 and 2 represent the most common hereditary neuropathies, characterized by distal weakness, muscle atrophy, diminished reflexes, and foot deformities, all of which lead to significant postural instability and gait disturbances [34]. Nutritional neuropathies, often associated with vitamin deficiencies, and immune-mediated forms such as polyneuropathy associated with monoclonal gammopathy or anti-MAG antibody neuropathy—commonly found in lymphoproliferative disorders—were also included. Additionally, paraneoplastic neuropathies linked to autoimmune responses secondary to neoplasms, as well as compressive neuropathies with focal involvement, were represented. Rare forms such as tomaculous neuropathy, a genetic disorder characterized by segmental myelin instability, were also reported.

Regardless of etiology, these neuropathic forms severely impair proprioception and peripheral sensory FB, thereby compromising automatic postural control and increasing the risk of falls. CIPN frequently presents with paresthesia, pain, diminished vibration and light touch sensitivity, and distal muscle weakness—all contributing to marked postural instability. In GBS, balance impairment is further exacerbated by motor dysfunction and the often slow or incomplete recovery of peripheral nerve connections, with predominant involvement of proximal musculature and difficulties in initiating and maintaining movement.

This etiological heterogeneity illustrates the rehabilitation challenges in polyneuropathies, with each subtype exhibiting distinct clinical patterns and functional responses in terms of postural balance and adaptability to neuromotor training interventions. Such diversity underscores the need for tailored rehabilitation strategies and accurate instrumental assessment methods.

### 4.2. Instruments Used

The platforms utilized in the studies reviewed operate based on common biomechanical principles, aiming to assess and train both static and dynamic balance. These devices measure center of pressure (CoP) displacements using integrated sensors and provide real-time visual or auditory FB, facilitating awareness and correction of postural imbalances.

Some platforms allow balance assessment under varied conditions, including eyes open and eyes closed—such as the SBM, Biodex Balance System, and Nintendo Wii Fit Balance Board—with the exception of the KAT 2000. The most commonly measured parameters include SA (postural oscillation), CoP velocity, LOS, and RFI. Exercise types range from maintaining the center of gravity within a target zone or following a predefined trajectory (e.g., SBM, KAT 2000) to interactive games that involve weight shifting and motor coordination (e.g., Wii Fit, Biodex).

While all these devices share the common goal of balance retraining, they differ in the nature of the stimulus provided: for instance, powered platforms can generate predictable external perturbations (e.g., sinusoidal oscillations) [30], which facilitate the training of anticipatory postural control (feedforward mechanisms); visual biofeedback platforms (e.g., SBM, KAT 2000) enhance conscious control; and interactive platforms (e.g., Wii Fit, Biodex) support implicit learning through engaging and motivational tasks. Each platform thus offers a specific intervention profile, yet all contribute to balance improvement by adaptively stimulating sensorimotor strategies.

All of these devices have generally shown high reliability across various published studies, supporting their potential validity in the evaluation and/or training of balance. For example, one study reported that balance training using the Biodex system significantly improved balance function in patients with neuropathy [35]. This system also appeared to be more effective than conventional programs in enhancing postural stability and motor function in children with cerebral palsy [36]. Another study demonstrated the feasibility of using the Nintendo Wii Balance Board in balance rehabilitation for patients with brain injuries [37]. Agmon et al. concluded that Wii Fit is a feasible and well-received balance training tool among older adults [38]. Moreover, the KAT was shown to be clinically effective in the rehabilitation of post-stroke patients, offering significant improvements [39].

Building upon these instrumental insights, the reviewed interventions shared several methodological similarities regarding balance training protocols, as detailed below.

### 4.3. Interventions—Training Programs

Balance training using stabilometric platforms generally consisted of maintaining an upright stance on a mobile or unstable surface that oscillates in controlled directions, requiring patients to continuously adjust their center of gravity. These sessions were assisted by real-time visual and/or auditory FB, enhancing the patient’s active engagement.

Training protocols and durations varied substantially across studies. Some applied short-term intensive regimens (e.g., daily sessions over a two-week period [30]), while others conducted programs over 4–6 weeks with a frequency of 2–3 sessions per week [27,28,29,32]. The duration of training sessions ranged from 30 to 60 min.

Many interventions followed the principle of gradual progression in difficulty, duration, and task repetition over several weeks, aiming to systematically increase the challenge for participants [31,32].

Kerim et al. [29] evaluated balance exercises recommended by the American College of Sports Medicine, both alone and in combination with the KAT 2000 system, and found that the combined approach did not offer additional benefits for dynamic balance. Nardone et al. [30] showed that improvements in balance and gait occurred regardless of the treatment sequence—whether exercises preceded platform training (EX–platform) or followed it (platform–EX), no significant differences were observed. Two articles [28,32] explored the efficacy of interactive game-based exercises and reported notable improvements in balance and functional performance, with superior outcomes compared to the group receiving only visual FB training.

Despite variability in the structure of interventions, most studies reported a trend toward significant improvements in static and/or dynamic balance control, as well as gait. The evidence suggests that the success of therapeutic outcomes is more closely related to how the training content is adapted—through difficulty level, sensory FB, and progression methods—rather than dosage alone. This observation aligns with the scientific literature; for instance, a 2024 meta-analysis on DPN [40] highlighted that multicomponent interventions produced the most substantial improvements in both static and dynamic balance parameters, regardless of program duration.

### 4.4. Posturographic Evaluation

All authors performed pre- and post-treatment evaluations using the devices employed in their respective interventions. The results obtained from posturographic assessments indicate a general trend of improvement in both static and dynamic postural control following the interventions analyzed. For example, in the study by Cammisuli et al. [27], he use of standardized SBM platform protocols (mCTSIB and LOS) led to a significant reduction in center of gravity sway velocity on unstable surfaces, suggesting enhanced sensory integration and increased postural stability under challenging conditions. These findings are consistent with those reported by Hosny et al. [28], who observed a decrease in the sensory integration index measured by the Biodex system, although no statistically significant intergroup differences were detected, likely due to the small sample size.

The effectiveness of KAT 2000-assisted exercises was supported by significant improvements in balance after four weeks of intervention [29], indicating that this type of training may facilitate proprioceptive re-education and optimize postural control. Although Nardone et al. [30] did not observe statistically significant changes under EO conditions, the trends noted under EC conditions may suggest partial reactivation of vestibular and somatosensory components in the absence of visual input.

Similarly, the case report by Hakim et al. [32], which utilized the Nintendo Wii Fit, revealed improvements in the LOS test, despite overall SOT scores remaining below normative levels. This discrepancy between LOS and SOT outcomes may indicate that certain components of dynamic balance respond more rapidly to interactive interventions, even when global sensory integration remains impaired. These findings are further supported by data from the VCFBT study [27], where significant improvements in LOS parameters were observed in 6 out of 7 patients, reinforcing the idea that visual FB and computer-assisted training may positively influence directional control and reaction time.

Our findings on postural control improvements are further corroborated by consistent evidence in the literature. Studies involving patients with DPN suggest that sensory-FB-based interventions—especially those combining reactive movement strategies with external visual FB—may help correct subtle postural dysfunctions, significantly contributing to improved balance control and reduced fall risk [19,41].

Sensor-based interactive balance training has demonstrated significant reductions in postural sway in patients with non-diabetic PN through adaptive visual FB and personalized exercise programs [42]. Similarly, visual-FB-based balance training on force platforms has proven effective in improving postural control among older adults [43], stroke survivors [44], and individuals with multiple sclerosis [45].

Regarding instrumental gait assessments, the TW and SUO tests have proven useful in analyzing multisensory integration during functional motor tasks. In the study by Cammisuli et al. [27], the VCFBT intervention led to a significant increase in TW speed in 6 out of 7 patients with CIPN (*p* = 0.011), along with a reduction in step width (*p* = 0.040), suggesting improved postural stability.

These findings suggest that visual FB-based interventions may favorably influence functional gait and dynamic balance parameters, although the effects appear to vary depending on the type of test employed and the complexity of the motor task involved. Comparable patterns of postural control improvement have been extensively documented in DPN, suggesting that FB-based balance training may rely on common sensorimotor mechanisms across different neuropathy etiologies rather than etiology-specific adaptations.

While posturographic data provide valuable insight into sensorimotor integration, clinical tests remain crucial for translating these improvements into functional outcomes.

### 4.5. Clinic Evaluations of Balance and Gait

Clinical functional evaluations of balance revealed significant improvements following the interventions applied in the analyzed studies, supporting the efficacy of sensory-FB-based strategies and platform-assisted training. The BBS, which assesses functional balance through a series of dynamic tasks (e.g., sit-to-stand transfers, turning, forward reaching), was frequently used as a reference tool. In the study by Cammisuli et al. [27], BBS scores significantly increased after the visual FB training program (*p* < 0.002), suggesting clinically meaningful improvements in functional balance. Similar results were reported by Kerim et al. [29], where both intervention groups showed significant gains (*p* < 0.01), although no statistically significant differences were observed between groups. In the case report by Hakim et al. [32], the BBS score increased from 28 to 34 out of 56 points, with the patient subsequently able to perform tasks such as maintaining stance with a narrow base of support and unilateral leg raises. Likewise, in the study by Albiol-Perez et al. [31], both patients showed improved BBS scores after completing 20 treatment sessions using the ABAR system. These findings support the hypothesis that technology-based interventions—particularly those incorporating sensory FB and dynamic components—can contribute effectively to functional recovery of postural control.

Gait disturbances represent one of the major consequences of polyneuropathies, manifested through reduced walking speed, variable step length, and instability during locomotion. Although not all included studies directly measured objective gait parameters, improvements in static and dynamic balance were indirectly reflected in safer and more efficient gait performance.

Beyond balance scores, several studies also highlighted significant improvements in functional mobility, using dynamic clinical tests such as the TUG and the TT. In the case report published by Hakim et al. [32], TUG time decreased from approximately 15 s to 11 s after six weeks of balance training, suggesting increased functional agility and improved dynamic gait control. Comparable results were reported by Kerim et al. [29], where TUG times decreased significantly in both analyzed groups (*p* < 0.01), with no relevant differences between the types of intervention (*p* = 0.05).

The TT, which evaluates both balance and gait components, was used as a complementary clinical tool in two of the analyzed studies [30,31]. Both investigations showed post-intervention score improvements, indicating enhanced performance in maintaining balance and executing gait in patients with PN.

Similar findings were reported by other authors who used stabilometric platforms for static and dynamic balance training in patients with DPN. In an experimental study conducted by Daut et al. [35] involving 38 DPN patients, a six-week balance training program on the Biodex Balance System significantly improved clinical outcomes: the mean BBS score increased from 42.7 to 48.9, and TUG time decreased from 17.5 to 13.6 s (both *p* < 0.001). Furthermore, Wii-based game therapy proved equally effective as standard proprioceptive exercise programs, both yielding clinically relevant improvements in a sample of 34 patients with DPN after six weeks of intervention (e.g., increased BBS scores, decreased TUG time) [46]. This convergence of results across diabetic and non-diabetic neuropathies supports the broader clinical applicability of feedback-based balance interventions, suggesting that such programs may effectively enhance stability and mobility regardless of underlying etiology.

The functional improvements observed through clinical testing have direct implications for one of the main therapeutic goals in neuropathic rehabilitation—reducing fall risk.

### 4.6. Risk of Fall

An essential objective of balance rehabilitation interventions in patients with peripheral neuropathy is the reduction in fall risk, given its significant impact on functional independence and quality of life. The findings from the reviewed studies indicate that most interventions—regardless of type (interactive gaming, visual FB, or conventional balance training)—were associated with a reduction in fall risk either through direct improvements in instrumentally quantified fall risk scores or via enhancement of clinically correlated parameters (e.g., TUG, BBS, TT). For instance, in a randomized study involving post-mastectomy patients with CIPN, both groups—one undergoing interactive exercise on a stabilometric platform and the other following a classical visual FB-based training—demonstrated a significant reduction in RFI after four weeks of intervention (*p* ≤ 0.001) [28]. These findings support the notion that both the content of the intervention and the patient’s active engagement in functional and sensorimotor tasks may play an important role in reducing imbalance and fall risk in this vulnerable population. An increase in ABC score—from 57.5% to 70.6%—reported in a case study [32] further indicates a meaningful improvement in confidence regarding balance during daily life activities, which potentially reflects a lower fall risk.

A recent meta-analysis reported that, in six out of sixteen studies, patients with DPN transitioned from moderate-to-high fall risk to low or no risk following various interventions, irrespective of the exact type of therapy. This underscores the overall effectiveness of interventions incorporating balance, gait, proprioceptive, and others [47]. Furthermore, among high-risk individuals with PN, structured balance programs have been identified as both safe and effective for reducing falls and improving lower limb strength and postural control [48]. Additionally, an observational study involving 146 patients with DPN found that each one-point increase in balance confidence (ABC score) was associated with a 9% decrease in fall likelihood (*p* < 0.001) [49].

In a recent randomized controlled trial published in 2025 [46], researchers compared the effects of Wii Fit-based training and proprioceptive exercises on balance and fall risk in patients with DPN. While no significant intergroup differences were noted post-intervention (*p* > 0.05), both groups showed significant intragroup improvements in BBS, Modified Fall Efficacy Scale (MFES), and TUG scores (*p* < 0.05), indicating that both interventions may exert clinically relevant effects in reducing fall risk. The consistency of these outcomes with studies conducted in DPN populations reinforces the notion that multisensory feedback and task-oriented balance training can effectively mitigate fall risk across different neuropathy etiologies.

These improvements in functional stability may extend beyond physical outcomes, positively influencing patients’ perceived well-being and daily independence.

### 4.7. Quality of Life

QoL is a critical outcome when evaluating the efficacy of rehabilitation interventions, particularly in patients with chronic conditions that impair daily functioning. Peripheral polyneuropathy, through its associated symptoms—such as imbalance, restricted mobility, fear of falling, and chronic pain—can significantly diminish QoL by reducing independence and increasing anxiety about daily activities. Among the studies included in this review, only a limited number directly assessed QoL using standardized questionnaires; however, the observed objective improvements suggest a potential positive impact on patients’ perceived well-being.

For instance, in the study conducted by Kerim et al. [29], significant improvements in QoL were observed in both intervention groups as measured by the NHP. However, in the group receiving only conventional balance training, no significant changes were reported in NHP components related to sleep and energy levels. In contrast, the group undergoing training with the KAT 2000 device showed greater improvements, suggesting an additional benefit of technology-assisted balance interventions. These findings support the hypothesis that multisensory interventions involving kinesthetic stimuli and real-time postural FB can positively affect not only functional balance but also subjective well-being domains such as sleep quality and energy.

Similarly, Dharwadkar et al. reported in 2024 significant QoL improvements in a cohort of 27 elderly patients with DPN, as measured by the EQ-5D-5L questionnaire, following four weeks of proprioceptive training. These improvements were accompanied by reductions in fall risk and enhanced balance performance (*p* = 0.001) [50]. In contrast, Venkataraman et al. found that a short-term, structured strength and balance training program did not significantly alter global health-related QoL (measured with SF-36 or EQ-5D-5L), though it led to sustained improvements in functional performance and balance confidence at six-month follow-up in patients with DPN [51]. Although evidence remains limited, these improvements in subjective well-being are consistent with reports from DPN rehabilitation studies, suggesting that enhanced balance control and confidence may translate into better perceived quality of life regardless of etiology.

Taken together, the evidence from the included studies highlights converging trends across balance, gait, fall risk, and quality of life domains, warranting an integrative interpretation.

### 4.8. Integrated Interpretation and Clinical Implications

When analyzed collectively, the findings indicate that platform-based balance training—especially when incorporating visual or auditory FB—consistently improves static and dynamic balance, functional mobility, and confidence in patients with non-diabetic PN. Improvements were reported across diverse neuropathic etiologies and device types, suggesting that the key therapeutic mechanism lies in the activation of sensory feedback and postural adjustment strategies.

Despite the heterogeneity of protocols and small sample sizes, the studies converge toward a consistent outcome: repeated, feedback-based, task-oriented practice improves functional balance and mobility. These findings highlight the potential role of stabilometric platforms as complementary rehabilitation tools, capable of promoting sensorimotor relearning and reducing fall risk in patients with PN. Even though the results are encouraging, several methodological limitations must be acknowledged and are discussed below.

### 4.9. Limitations of the Study

It is important to acknowledge several limitations that may influence the interpretation of the present findings. First, the body of evidence available for non-diabetic peripheral neuropathies is still very limited: only six studies met the eligibility criteria, and several of them were pilot trials, case series, or small-sample interventions (*n* < 20). This reduced statistical power and prevented us from conducting a quantitative synthesis or exploring dose–response relationships. Second, the high heterogeneity among the included studies complicates direct comparisons and precludes meaningful aggregated quantitative analysis. Non-diabetic PN encompasses a wide range of etiologies (e.g., toxic, autoimmune, hereditary), and patients may exhibit varying levels of severity and distinct functional deficits.

Additionally, the rehabilitation protocols differed markedly with respect to the type of platform (SBM, Biodex, KAT, Wii), feedback modality (visual vs. interactive/gamified), session frequency (daily vs. 2–3/week), and program length (2–6 weeks). Because of this, the present review could only describe trends and not identify an optimal training dose or a standardized progression model. Not all studies assessed the same outcome categories; for instance, some omitted gait parameters or Qol measures, resulting in gaps in the comprehensiveness and comparability of findings.

Another key limitation is the lack of long-term follow-up data. Most studies assessed participants immediately post-intervention (except for the case that had a 6-month follow-up without decline [32]), leaving unclear whether the observed improvements are sustained over months or years, or whether periodic booster sessions might be required to maintain benefits. Finally, only a minority of the included studies were large-scale randomized controlled trials (RCTs), and we included only articles available in English and indexed in the databases specified in the Methods; unpublished data, grey literature, or non-English studies may exist and could slightly modify the overall picture of available evidence. This highlights the need for further high-quality research before definitive conclusions can be drawn or evidence-based clinical guidelines can be established.

### 4.10. Future Directions

Future research should aim to overcome these limitations through well-designed, adequately powered, and preferably multicenter randomized controlled trials that focus specifically on non-diabetic PN populations. Intervention protocols should be standardized and fully described (platform type, feedback mode, session duration, progression criteria) to enable replication and direct comparison between studies and centers. It would also be useful to compare platform-based training versus conventional balance/proprioceptive therapy and versus combined programs, in order to determine the true added value of technology-assisted interventions.

Furthermore, upcoming studies should adopt a core set of outcome measures that includes (i) instrumental/posturographic parameters (CoP sway, LOS, sensory integration indices); (ii) validated clinical functional tests (BBS, TUG, TT); and (iii) patient-centered outcomes such as fall-related self-efficacy and health-related quality of life. Follow-up assessments at ≥6 and preferably 12 months are needed to clarify whether improvements are maintained over time or whether booster sessions on the platform are necessary. Finally, given the costs of stabilometric and VR-based systems, cost-effectiveness and implementation studies would help determine feasibility in routine clinical practice and in different rehabilitation settings.

## 5. Conclusions

The use of stabilometric platforms appears to represent an innovative and promising strategy for the rehabilitation of balance disorders in patients with non-diabetic PN. Although the available evidence remains limited and methodologically heterogeneous, it suggests potential improvements in postural control (both static and dynamic), a reduction in fall risk, as well as functional benefits and possible effects on QoL. However, the variability in etiology, interventions, and assessment methods limits the generalizability of the conclusions. Large-scale, rigorous randomized clinical trials with standardized protocols and long-term follow-up are needed to confirm the efficacy and specific value of stabilometric training in comparison with conventional rehabilitation.

## Figures and Tables

**Table 3:** Synthesis of the main characteristics and outcomes of the studies included in the systematic review. The table summarizes key methodological aspects (sample size, age, and type of peripheral neuropathy), intervention parameters (type of stabilometric or force platform, frequency, and duration of training), and the principal outcome measures related to balance, gait, risk of fall, and quality of life. See Abbreviations section for full term definitions.

Authors	Sample	Age (Average, Standard Dev.)	Type of PN	Platform—Training Program	Frequency and Duration	Outcomes
**Cammisuli et al. (2016)** [27]	*N* = 7	64.14 ± 9.91	CIPN	**The Smart Balance Master (SBM)** - The exercises chosen were rhythmic weight shifting (RWS) (4 min and was repeated four times with a 1 min rest in-between repetitions, on stable and unstable platform), TW (forward and backward walk was continued for 4 min. The exercise was repeated twice with a 1 min interval between repetitions), and SUO (4 min and was repeated twice with a 1 min interval between repetitions).	60 min 3 ses/week 4 weeks	↓ COG sway velocity (on the unstable surface with EO and EC) ↑ LOS ↑ TW ↑ BBS
**Hosny et al.** **(2022)** [28]	group (A): *N* = 15 group (B): *N* = 15	A: 45.42 ± 5.26 B: 47.60 ± 4.92	CIPN	**Biodex Balance System** A: Received game-based training—The patient played a balance game on the Biodex system, shifting weight to kick a virtual ball toward a target. B: Received visual FB training—The patient adjusted foot position to maintain balance on a moving platform, keeping a cursor centered on a screen displaying limits of stability.	30 min 3 ses/week 4 weeks	↓ mTNS—A > B ↓ RFI—A > B ↓ SI—no difference
**Kerim et al.** **(2018)** [29]	group 1: *N* = 30 group 2: *N* = 30	1: 75.70 ± 6.71 2: 75.43 ± 6.54	PN	**Kinesthetic Ability Trainer** (KAT 2000) 1: Balance training included progressive postures to narrow the base of support, dynamic movements to shift the center of gravity, exercises targeting postural muscles and sensory-challenging tasks. 2: All patients performed balance exercises, supplemented with KAT 2000 training. – Static training: Patients stood still with arms crossed, maintaining balance and keeping a red X centered on the screen. – Dynamic training: Patients followed the red X through various patterns (circles, squares, figure-eights) for 30 s, repeated three times. Both groups also did a 10 min warm-up and cool-down (including stretching) before and after training.	45 min 3 ses/week 4 weeks	↑ KAT 2000 static and dynamic scores—no difference ↑ BBS—no statistical difference ↓ TUG—no statistical difference ↑ quality of life—in both groups (except NHP sleep and NHP energy level scores in group 1)
**Nardone et al.** **(2010)** [30]	neuropathy: *N* = 19 vestibular deficit: *N* = 14 EX-powered platform group: *N* = 16 platform-EX group: *N* = 17	58.4 ± 17.4	PN/vestibular disorder	not mentioned EX-platform group: Began with disease-specific balance exercises (modified Frenkel exercises), then switched to powered platform training. Platform-EX group: Started with powered platform training, then continued with balance exercises.	30 min 2 ses/day 5 days/week 2 weeks	↑ postural control in both groups (vestibular patients > neuropathic patients) ↓ SA—no difference ↑ subjective score of stability—no difference ↑ TT—no difference
**Albiol-Pérez et al.** **(2013)** [31]	*N* = 2	33, 54	GBS	**Active Balance Rehabilitation (ABAR) system** - For static balance, the system requires patients to perform weight shifts in both sitting and standing positions, targeting medio-lateral and antero-posterior directions. For dynamic balance, ABAR prompts the patient to carry out various movements while standing, such as stepping onto the Wii Balance Board or performing sit-to-stand transitions.	30 min 20 sessions	↑ ART ↑ BBS ↑ TT ↑ SEQ
**Hakim et al. (2015)** [32]	*N* = 1	76	PN (unknown etiology)	**Nintendo Wii Fit gaming system** - Patient performed balance and strengthening games on the Nintendo Wii Fit using a gait belt and appropriate supervision for safety. Activities were selected from Wii Sports and Wii Fit programs based on his specific functional, strategic, and impairment-level needs.	60 min 2 ses/week 6 weeks	↑ LOS SOT—same ↑ BBS ↓ TUG ↑ ABC ↑ CST

↑ indicates increase/improvement; ↓ indicates decrease/reduction.

## Data Availability

All data supporting the findings of this study are available in the original articles included in the systematic review, which are cited in the reference list. No new data were generated. Further inquiries can be directed to the corresponding authors.

## Supplementary Materials

The following supporting information can be downloaded at: https://www.mdpi.com/article/10.3390/jcm14228049/s1, PRISMA 2020 Main Checklist; Table S1: Number of articles retrieved from each database using the Boolean search string.

## Abbreviations

The following abbreviations are used in this manuscript:

PN	Peripheral neuropathy
VR	Virtual reality
FB	Feedback
PRISMA	Preferred Reporting Items for Systematic Reviews and Meta-Analyses
DPN	Diabetic peripheral neuropathy
GBS	Guillan–Barré syndrome
HMSN	hereditary motor–sensory neuropathies
CIDP	Chronic inflammatory demyelinating polyneuropathy
MMN	Multifocal motor neuropathy
RCTs	Randomized controlled trials
PEDro	Physiotherapy Evidence Database
RoB 2	Cochrane Risk of Bias 2
ROBINS-I V2	Risk Of Bias in Non-randomized Studies of Interventions, Version 2
VCFBT	Visual computer-feedback balance training
CIPN	Chemotherapy-induced peripheral neuropathy
SBM	Smart Balance Master
EO	Eyes open
EC	Eyes closed
COG	Center of gravity
LOS	Limits of Stability
TW	Tandem Walk
BBS	Berg Balance Scale
SUO	Step up/over
mTNS	Modified total neuropathy score
M-CTSIB	Modified clinical test of sensory integration and balance
KAT	Kinesthetic Ability Trainer
QoL	Quality of life
TUG	Timed Up and Go
NHP	Nottingham Health Profile
EX	Exercice
SA	Sway area
ABAR	Active Balance Rehabilitation
ART	Anterior Reach Test
TT	Tinetti Test
SEQ	Suitability Evaluation Questionnaire
SOT	Sensory Organization Test
MXE	Maximum Endpoint Excursion
MCT	Motor Control Test
ADT	Adaptation Test
CST	Chair Stand Test
ABC	Activity-Specific Balance Confidence
RWS	Rhythmic weight shifting
RFI	Risk of falling index
SI	Sensory integration
CMT	Charcot–Marie–Tooth
CoP	Center of pressure
MFES	Modified Fall Efficacy Scale
