# Supplementary material for: Is Balance Training Using the Stabilometric Platforms Integrating Virtual Reality and Feedback Effective for Patients with Non-Diabetic Peripheral Neuropathy?—A Systematic Review
Source: J Clin Med. 2025 Nov 13;14(22):8049. doi: 10.3390/jcm14228049 (PMC12653828; doi:10.3390/jcm14228049)
Supplement: Supplementary file 1 [file jcm-14-08049-s001.zip › Supplementary Table S1.pdf]

Table S1. Number of articles retrieved from each database using the Boolean search string.

Databases	Boolean Search String	Filters Applied	Number of Results
**PubMed**	(("peripheral neuropathy"[MeSH Terms] OR "peripheral neuropathy"[Title/Abstract]) AND ("stabilometric platform"[Title/Abstract] OR "force platform"[Title/Abstract] OR "postural control"[Title/Abstract] OR "balance training"[Title/Abstract] OR "postural balance"[Title/Abstract]) AND ("rehabilitation"[MeSH Terms] OR "rehabilitation"[Title/Abstract] OR "physical therapy"[Title/Abstract]))	Humans, English, Adult	6 results
**Web of Science**	TS=("peripheral neuropathy") AND TS=("stabilometric platform" OR "force platform" OR "postural control" OR "balance training" OR "postural balance") AND TS=("rehabilitation" OR "physical therapy")	English	1 results
**ScienceDirect**	("peripheral neuropathy") AND ("stabilometric platform" OR "force platform" OR "postural control" OR "balance training" OR "postural balance") AND ("rehabilitation" OR "physical therapy")	Research articles	141 results
**SCOPUS**	TITLE-ABS-KEY(("peripheral neuropathy") AND ("stabilometric platform" OR "force platform" OR "postural control" OR "balance training" OR "postural balance") AND ("rehabilitation" OR "physical therapy"))	English	3 results
**Springer Nature Link**	("peripheral neuropathy") AND ("stabilometric platform" OR "force platform" OR "postural control" OR "balance training" OR "postural balance") AND ("rehabilitation" OR "physical therapy")	English	881 results
**Cochrane Library**	("peripheral neuropathy") AND ("stabilometric platform" OR "force platform" OR "postural control" OR "balance training" OR "postural balance") AND ("rehabilitation" OR "physical therapy")	Trials & Reviews	3 results
**WILEY Online Library**	("peripheral neuropathy") AND ("stabilometric platform" OR "force platform" OR "postural control" OR "balance training" OR "postural balance") AND ("rehabilitation" OR "physical therapy")	English	75 results
**TOTAL**			**1110 results**
